# Perceptions and Ethical Concerns Regarding the Use of Artificial Intelligence in Mental Healthcare Among the Mental-Health Workforce: A Cross-Sectional Study

**DOI:** 10.7759/cureus.102623

**Published:** 2026-01-30

**Authors:** Nikita Saini, Arjun Segu, Matthew Yu, Rohit Mishra

**Affiliations:** 1 Dentistry, Ranjhi Government Hospital, Jabalpur, IND; 2 Research, Dougherty Valley High School, San Ramon, USA; 3 Research, Bridgewater-Raritan Regional High School, Bridgewater, USA; 4 Periodontics and Implantology, Hitkarini Dental College and Hospital, Jabalpur, IND

**Keywords:** artificial intelligence, cross-sectional survey, ethical awareness, mental healthcare, mental health professionals, united states

## Abstract

Introduction: Artificial intelligence (AI) is increasingly incorporated into mental healthcare, offering opportunities to improve diagnostic accuracy, service accessibility, and administrative efficiency. However, effective implementation depends on the mental health workforce's awareness, perceptions, and ethical concerns related to AI. Evidence regarding these factors remains limited within diverse practice settings in the United States (US).

Methods: A descriptive cross-sectional survey was conducted among mental health professionals and trainees practicing in two US states: California and New Jersey. Data were collected using a structured, self-administered online questionnaire adapted from the validated Shinners Artificial Intelligence Perception (SHAIP) scale and expanded to include an ethical concern domain. The survey assessed demographic characteristics, AI awareness, perceptions, ethical concerns, and barriers and facilitators to AI adoption. Statistical analyses were performed using Statistical Product and Service Solutions (SPSS, version 26; IBM SPSS Statistics for Windows, Armonk, NY). Descriptive statistics summarized participant characteristics, while inferential analyses, including chi-square, Mann-Whitney U, and Kruskal-Wallis tests, were used to examine bivariate differences in AI-related outcomes across participant characteristics, with statistical significance set at p < 0.05.

Results: A total of 220 mental health professionals and trainees participated, representing psychiatry, psychology, and allied mental health disciplines. Although 63.2% reported formal training in AI, only 39.6% reported using AI-assisted systems in clinical practice. Overall awareness and perceptions of AI were positive, with mean scores ranging from 3.45 to 3.75 across awareness and perception domains. Participants endorsed AI's potential to enhance diagnostic accuracy, reduce administrative workload, and improve access to mental health services. Ethical concerns were prominent, particularly regarding potential bias in clinical decision-making (mean = 3.81) and data privacy and security (mean = 3.72). Familiarity with AI concepts, years of clinical experience, and formal AI training were significantly associated with awareness, perception, and ethical-awareness scores (p < 0.05), while age and gender were not.

Conclusion: Mental health workforce demonstrated favorable attitudes toward AI in mental healthcare but reported limited real-world adoption and substantial ethical concerns. These findings underscore the need for targeted education, robust ethical frameworks, and practical training to bridge the gap between AI awareness and responsible clinical implementation.

## Introduction

Mental health is a fundamental component of overall health, encompassing emotional, psychological, and social well-being and influencing how individuals cope with stress, function in daily life, and contribute to society [1]. Mental health disorders - including depression, anxiety disorders, bipolar disorder, substance use disorders, and schizophrenia - constitute a major public health burden in the United States, affecting millions of individuals and placing substantial demands on healthcare systems [2].

In response to growing clinical workloads, workforce shortages, and increasing complexity of care, digital innovations, particularly artificial intelligence (AI), are being progressively integrated into mental healthcare delivery. AI broadly refers to computational systems capable of performing tasks that traditionally require human intelligence, such as learning, reasoning, pattern recognition, and decision-making [3]. Within healthcare, AI applications include machine learning algorithms, natural language processing, predictive analytics, and automated decision-support systems, all of which aim to improve efficiency, accuracy, and personalization of care [4]. In mental healthcare, AI has shown promise in screening and early detection of psychiatric conditions, symptom monitoring, treatment planning, administrative automation, and expansion of care access through digital platforms and conversational agents [5].

Despite these advancements, the successful integration of AI into mental healthcare is not solely dependent on technological capability. The mental health workforce (professionals and trainees) plays a pivotal role in determining whether AI tools are adopted, trusted, and ethically implemented in clinical practice [6]. Prior studies suggest that clinicians often express cautious optimism toward AI, recognizing its potential to support diagnostic accuracy, reduce administrative burden, and enhance service accessibility while simultaneously emphasizing that AI should complement rather than replace human judgment and therapeutic relationships [7,8].

Ethical considerations represent a particularly salient concern in the application of AI to mental healthcare. Issues related to data privacy, algorithmic bias, transparency, accountability, and the preservation of patient autonomy are amplified in mental health settings due to the sensitive nature of patient information and the centrality of trust in therapeutic alliances [9,10]. Biased datasets may disproportionately affect vulnerable populations, while opaque decision-making processes can undermine clinician confidence and patient acceptance [11]. Consequently, ethical concerns and attitudes toward governance among the mental health workforce are increasingly recognized as important for responsible AI adoption.

Emerging evidence indicates that clinicians' awareness, perceptions, and ethical attitudes toward AI may be influenced by demographic and professional factors such as years of clinical experience, level of digital literacy, prior exposure to AI training, and professional discipline [12,13]. However, findings across studies remain inconsistent, and much of the existing literature focuses on general healthcare professionals rather than those working specifically in mental health contexts. Moreover, United States (US)-based data examining both perceptions of AI and ethical awareness among mental health professionals and trainees across diverse practice settings remain limited.

Given the accelerating incorporation of AI into mental healthcare systems in the US, there is a clear need to better understand how mental health workforce perceives these technologies and how ethically prepared they feel to engage with them in clinical practice. Addressing this gap is critical for informing targeted education, policy development, and implementation strategies that promote safe, equitable, and effective use of AI in mental healthcare.

Therefore, the present study aimed to assess awareness of AI, perceptions toward its use in mental healthcare, and ethical concerns related to AI among the mental health workforce, including licensed professionals and trainees, practicing in two US states (California and New Jersey). The study further explored bivariate differences in AI-related awareness, perceptions, and ethical concern scores across selected demographic and professional characteristics, including clinical experience, prior AI training, and self-reported familiarity with AI concepts.

## Materials and methods

Study design

A descriptive cross-sectional study was conducted to assess awareness, perceptions, and ethical considerations related to the use of AI among mental health professionals and trainees. Ethical approval was obtained from the Institutional Ethical Committee (HDCH-IEC). The study was granted exemption under the minimal-risk category (IEC reference number: HDCH-IEC 24/2025). Informed consent was obtained from all participants prior to enrollment.

Study setting

The study was carried out in two US states: California and New Jersey. Participants were recruited from diverse professional environments, including hospitals and mental health institutions, academic and teaching institutions, private practices, and community mental health centers.

Study population

The study population comprised licensed mental health professionals and trainees (including psychology students) involved in mental health service delivery or clinical training. These included psychiatrists, clinical psychologists, counselling psychologists, psychiatric nurses, psychiatric social workers, and psychology students currently engaged in clinical training or practice.

Inclusion and exclusion criteria

Participants were eligible for inclusion if they were aged 20 years or older, were qualified mental health professionals or currently in training, practiced or trained in California or New Jersey, and provided informed consent electronically. Individuals not affiliated with the mental health field, those practicing outside the selected regions, and responses that were incomplete or duplicated were excluded from the study.

Study instrument

Data were collected using a structured, self-administered online questionnaire developed using Google Forms. The instrument was designed to assess awareness, perceptions, ethical concerns, and practical experiences related to the use of AI in mental healthcare among the mental health workforce, including licensed professionals and trainees. Items assessing perceptions of AI and its potential impact on clinical care and professional roles were adapted and conceptually informed by the Shinners Artificial Intelligence Perception (SHAIP) questionnaire, a validated instrument developed to measure healthcare professionals' perceptions and preparedness toward AI implementation. The original SHAIP instrument focuses on clinicians' views regarding the clinical utility of AI, its perceived role in professional practice, and readiness for AI integration [1]. In the present study, selected SHAIP-derived items were adapted in wording and contextualized to mental healthcare settings while retaining the core conceptual intent of the original framework.

Additional items assessing general awareness of AI, ethical concerns, and attitudes toward governance (including issues related to privacy, algorithmic bias, informed consent, and the need for ethical guidelines), barriers and facilitators to AI adoption, and clinical use of AI tools were newly developed by the authors, as these domains are not included in the original SHAIP instrument. The development of these items was informed by a review of existing literature on AI in healthcare and mental health and aimed to capture context-specific ethical and practical considerations relevant to mental health practice.

The final questionnaire comprised 29 items covering participant information and consent, demographic and professional characteristics, general awareness of AI, perceptions toward AI in mental healthcare (adapted and conceptually informed by SHAIP), ethical concerns related to AI, perceived barriers and facilitators to AI adoption, and clinical use of AI tools, including open-ended reflections. Items assessing awareness, perceptions, and ethical concerns were rated on a five-point Likert scale ranging from strongly disagree (1) to strongly agree (5). Barriers and facilitators were assessed using predefined multiple-response options, and open-ended items were included to allow participants to elaborate on their experiences and perspectives regarding responsible AI use in mental health practice. The questionnaire required approximately 8-10 minutes to complete. In this study, the term “AI tools” refers to software or digital systems incorporating machine learning, natural language processing, or automated decision-support functionalities used for clinical, administrative, or supportive purposes within mental healthcare settings.

Validity and reliability

Content and face validity of the questionnaire were reviewed by subject experts in psychiatry, psychology, and ethics to ensure clarity, relevance, and contextual appropriateness. A pilot test was conducted among 15 mental health professionals to assess comprehension and completion time. Based on feedback obtained during pilot testing, minor modifications were made, including refinement of item wording for clarity, elimination of redundancy, and contextual adaptation of selected items to mental healthcare practice. Internal consistency reliability was assessed using Cronbach's alpha, which demonstrated good reliability across domains, with values of 0.82 for awareness, 0.80 for perception, and 0.84 for ethical concern items.

Data collection procedure

The survey link was disseminated electronically through professional networks, institutional mailing lists, and direct professional contacts. Potential participants were approached between October and December 2025, primarily via professional mailing lists, institutional contacts, and online professional networks targeting mental health professionals and trainees in California and New Jersey. Participation was voluntary, and informed consent was obtained electronically prior to accessing the questionnaire. Responses were anonymous, and no personally identifiable information was collected. Duplicate submissions were restricted through platform settings. The survey remained open for a period of six weeks, during which reminder notifications were issued to improve response rates.

Sample size calculation

The sample size calculation was based on the primary objective of examining differences in awareness, perception, and ethical concern scores across key demographic and professional characteristics. Sample size was calculated a priori using G*Power software based on a chi-square goodness-of-fit model to ensure adequate power for detecting group-wise differences across key categorical characteristics in this exploratory study. Assuming an effect size (w) of 0.3, an alpha error probability of 0.05, statistical power of 0.95, and three degrees of freedom, the minimum required sample size was estimated to be 191 participants. The analysis yielded a noncentrality parameter (λ) of 17.19 and a critical chi-square value of 7.81, with an actual power of 0.95. A total of 220 complete responses were obtained and included in the final analysis, exceeding the minimum required sample size.

Statistical analysis

Data were analyzed using Statistical Product and Service Solutions (SPSS, version 26; IBM SPSS Statistics for Windows, Armonk, NY). Descriptive statistics were computed as frequencies, percentages, means, and standard deviations. Given the ordinal nature of Likert-scale responses and the absence of normal distribution, non-parametric statistical methods were applied. Group-wise comparisons of awareness, perception, and ethical concern scores across multiple categories were performed using the Kruskal-Wallis H test, while comparisons between two groups were analyzed using the Mann-Whitney U test. Although the Kruskal-Wallis test reports a chi-square (χ²) statistic, this represents the non-parametric H statistic and not Pearson's chi-square test.

The chi-square test was used only for associations between nominal categorical variables. Bonferroni correction was applied for post-hoc pairwise comparisons where appropriate. Given the exploratory nature of the study and the large number of subgroup comparisons, no global adjustment across all analyses was performed, and findings should be interpreted cautiously due to the potential for type I error.

## Results

Participant characteristics

A total of 220 mental health professionals and trainees participated in the study. The largest age groups were 30-39 years (30.9%) and 40-49 years (30.0%). Gender distribution was nearly balanced, with 50.0% of participants identifying as female and 45.9% as male, while 4.1% preferred not to disclose gender. Counselling psychologists constituted the largest professional group (27.3%), followed by clinical psychologists (22.7%), psychiatric nurses (19.1%), psychiatric social workers (11.4%), psychology students (10.5%), and psychiatrists (9.1%).

Most participants reported 5-10 years of clinical experience (48.6%), followed by 11-20 years (21.4%) and less than five years (20.9%). Regarding work setting, the majority were employed in academic or teaching institutions (36.8%) and hospitals or mental-health institutions (36.4%), with smaller proportions working in private practice (12.7%) and community mental health centers (12.7%). Geographically, 57.7% of participants were based in California, and 42.3% in New Jersey (Table 1).

**Table 1:** Demographic characteristics of participants (n = 220). n (%) means the number of participants and their percentage.

Characteristic	n (%)
Age (years)	
20-29	37 (16.82)
30-39	68 (30.91)
40-49	66 (30.00)
50-59	41 (18.64)
≥60	08 (3.64)
Gender	
Male	101 (45.91)
Female	110 (50.00)
Prefer not to say	09 (4.09)
Professional Role	
Psychiatrist	20 (9.09)
Clinical Psychologist	50 (22.73)
Counselling Psychologist	60 (27.27)
Psychiatric Nurse	42 (19.09)
Psychiatric Social Worker	25 (11.36)
Psychology Student	23 (10.45)
Work Setting	
Private practice	28 (12.73)
Hospital/Mental health institution	80 (36.36)
Academic/Teaching institution	81 (36.82)
Community mental health centre	28 (12.73)
Others	03 (1.36)
Years of Clinical Experience	
None	16 (7.27)
Less than 5 years	46 (20.91)
5–10 years	107 (48.64)
11–20 years	47 (21.36)
More than 20 years	04 (1.82)
Employment Location	
California	127 (57.73)
New Jersey	93 (42.27)

Awareness and exposure to AI

With respect to exposure to AI, 63.2% of participants reported having received formal training related to AI in healthcare. Approximately half of the respondents (50.5%) reported current use or interaction with AI tools in their professional setting, while 42.7% reported no such use and 6.8% were unsure. In terms of familiarity with AI concepts in mental healthcare, most participants described themselves as somewhat familiar (40.9%) or moderately familiar (38.2%). A smaller proportion reported being highly familiar (9.5%), while 11.4% indicated that they were not familiar with AI concepts in mental healthcare (Table 2).

**Table 2:** Distribution of the study subject's responses towards artificial intelligence (n = 220). n (%) means the number of participants and their percentage.

Items	Response	n (%)
Formal training on AI in healthcare	Yes	139 (63.18)
	No	81 (36.82)
Current use or interaction with AI tools in professional setting	Yes	111 (50.45)
	No	94 (42.73)
	Unsure	15 (6.82)
Familiarity with AI concepts in mental healthcare	Not familiar	25 (11.36)
	Somewhat familiar	90 (40.91)
	Moderately familiar	84 (38.18)
	Highly familiar	21 (9.55)

General awareness of AI

Overall awareness of AI among participants was moderate to high, with mean scores ranging from 3.45 to 3.66 across awareness-related items. The highest mean score was observed for the statement that AI tools can reduce administrative workload for clinicians (mean = 3.66 ± 1.02). Awareness that AI is used in various areas of healthcare demonstrated the lowest mean score (mean = 3.45 ± 0.92). More than half of the respondents agreed or strongly agreed that AI can assist in the early detection of mental health conditions and that they had encountered AI-based tools relevant to mental health practice (Table 3).

**Table 3:** Frequency distribution of responses to general awareness statements on artificial intelligence showing the number of participants n (%), mean, and standard deviation (SD).

Statements	Responses					
	Strongly Disagree, n (%)	Disagree, n (%)	Neutral, n (%)	Agree, n (%)	Strongly Agree, n (%)	Mean ± SD
I am aware that AI is used in various areas of healthcare.	07 (3.18)	21 (9.55)	82 (37.27)	86 (39.09)	24 (10.91)	3.45 ± 0.92
I have encountered AI-based tools relevant to mental health.	04 (1.82)	24 (10.91)	63 (28.64)	87 (39.55)	42 (19.09)	3.63 ± 0.97
AI can assist in the early detection of mental-health conditions.	05 (2.27)	19 (8.64)	75 (34.09)	85 (38.64)	36 (16.36)	3.58 ± 0.94
AI tools can reduce administrative workload for clinicians.	07 (3.18)	19 (8.64)	64 (29.09)	82 (37.27)	48 (21.82)	3.66 ± 1.02

Perceptions toward AI in mental healthcare

Participants demonstrated generally positive perceptions toward AI in mental healthcare, with mean scores ranging from 3.61 to 3.75. The highest level of agreement was observed for the statement that AI can improve accessibility of mental health services (mean = 3.75 ± 1.01). More than 60% of respondents agreed or strongly agreed that AI can enhance diagnostic accuracy and support early prediction of relapse or risk behaviors, and that AI-based virtual assistants can provide preliminary support to clients. Overall, perceptions toward the clinical utility of AI were favorable across professional groups (Table 4).

**Table 4:** Distribution of participants’ responses to perception statements on artificial intelligence in mental healthcare showing number of respondents n (%), mean scores, and standard deviation (SD).

Statements	Responses					
	Strongly Disagree, n (%)	Disagree, n (%)	Neutral, n (%)	Agree, n (%)	Strongly Agree, n (%)	Mean ± SD
AI can enhance diagnostic accuracy.	05 (2.27)	16 (7.27)	66 (30.00)	92 (41.82)	41 (18.64)	3.67 ± 0.94
AI tools can support early prediction of relapse or risk behaviours.	04 (1.82)	22 (10.00)	71 (32.27)	82 (37.27)	41 (18.64)	3.61 ± 0.96
AI-based virtual assistants can provide preliminary support to clients.	03 (1.36)	13 (5.91)	68 (30.91)	94 (42.73)	42 (19.09)	3.72 ± 0.87
AI can improve accessibility of mental-health services.	06 (2.73)	16 (7.27)	62 (28.18)	79 (35.91)	57 (25.91)	3.75 ± 1.01

Ethical concerns and attitudes related to AI

Participants reported substantial ethical concerns and strong endorsement of ethical safeguards related to the use of AI in mental healthcare. Concerns related to potential bias in diagnosis or clinical decision-making received the highest mean score (mean = 3.81 ± 0.96). Concerns regarding privacy and data security were also prominent, with 59.6% of respondents agreeing or strongly agreeing with this statement (mean = 3.72 ± 0.99). A majority of participants emphasized the need for clear ethical guidelines governing AI use in mental healthcare (mean = 3.64 ± 1.00). Additionally, 66.4% of respondents agreed or strongly agreed that informed consent should be obtained prior to the use of AI tools in mental health practice (Table 5).

**Table 5:** Distribution of participants' responses to ethical awareness and concern statements regarding artificial intelligence in mental healthcare showing number of respondents n (%), mean scores, and standard deviation (SD).

Statements	Responses					
	Strongly Disagree, n (%)	Disagree, n (%)	Neutral, n (%)	Agree, n (%)	Strongly Agree, n (%)	Mean ± SD
AI use raises concerns regarding privacy and data security.	01 (0.45)	26 (11.82)	62 (28.18)	76 (34.55)	55 (25.00)	3.72 ± 0.99
Clear ethical guidelines are needed for AI in mental healthcare.	07 (3.18)	16 (7.27)	74 (33.64)	75 (34.09)	48 (21.82)	3.64 ± 1.00
AI may introduce bias in diagnosis or decision-making.	03 (1.36)	16 (7.27)	60 (27.27)	81 (36.82)	60 (27.27)	3.81 ± 0.96
Informed consent should be obtained before using AI tools.	05 (2.27)	21 (9.55)	48 (21.82)	94 (42.73)	52 (23.64)	3.76 ± 0.99

Barriers and facilitators to AI adoption

Lack of training was identified as the most frequently reported barrier to AI adoption, cited by 60.5% of participants. Ethical or legal uncertainty (53.2%) and high cost or limited resources (49.1%) were also commonly reported barriers. Other barriers included resistance to change (31.4%) and lack of awareness (30.5%). Factors perceived to facilitate AI adoption included the establishment of clear guidelines and regulations (58.6%), availability of more clinical research and evidence (56.4%), and development of user-friendly AI tools (46.4%). Professional education and training were identified as facilitators by 31.8% of respondents. Despite these perceived facilitators, only 39.6% of participants reported using any AI-assisted system in their clinical work (Table 6).

**Table 6:** Distribution of reported barriers to and facilitators of artificial intelligence adoption in mental healthcare and current use of AI-assisted systems, expressed as number of respondents n and percentage (%).

Barriers & facilitators	Responses	n (%)
Barriers to AI adoption	Lack of awareness	67 (30.45)
	Lack of training	133 (60.45)
	Ethical or legal uncertainty	117 (53.18)
	High cost/limited resources	108 (49.09)
	Resistance to change	69 (31.36)
Helping in adopting AI in mental-health practice	Professional education and training	70 (31.82)
	Clear guidelines and regulations	129 (58.64)
	More clinical research and evidence	124 (56.36)
	User-friendly AI tools	102 (46.36)
	Integration into existing workflows	44 (20.00)
Use of any AI-assisted system in clinical work	Yes	87 (39.55)
	No	133 (60.45)

Bivariate differences in general awareness scores across participant characteristics

Table 7 presents the comparison of mean general awareness scores of AI across various participant characteristics using non-parametric statistical tests. Mean awareness scores were comparable across age groups, with values ranging from 3.43 ± 1.09 in the 20-29-year age group to 3.70 ± 0.86 in the 40-49-year age group, and these differences were not statistically significant (χ² = 6.827; p = 0.145). Similarly, no significant differences in awareness scores were observed based on gender (χ² = 1.417; p = 0.492), professional role (χ² = 3.289; p = 0.655), work setting (χ² = 6.263; p = 0.180), or employment location (U = 88484.000; p = 0.090). In contrast, statistically significant differences in general awareness scores were observed with respect to years of clinical experience (χ² = 25.950; p < 0.001).

**Table 7:** Bivariate differences in general awareness scores across participant characteristics. All reported comparisons represent unadjusted, bivariate analyses. Values are expressed as mean ± standard deviation (SD). Comparisons across multiple groups were performed using the Kruskal–Wallis H test (#), while two-group comparisons were analyzed using the Mann–Whitney U test. χ² indicates the Kruskal–Wallis test statistic; df denotes degrees of freedom; U denotes the Mann–Whitney U statistic. Pearson’s chi-square test was not applied to ordinal Likert-scale outcome variables. A p-value < 0.05 was considered statistically significant. Additionally, findings related to participants with more than 20 years of clinical experience should be interpreted cautiously due to the very small subgroup size (n = 4).

Characteristic	Category	Mean ± SD	χ²/U value	df	p value	Interpretation
Age (years)	20–29	3.43 ± 1.09	6.827	4	0.145	Not significant
	30–39	3.56 ± 0.99	6.827	4	0.145	Not significant
	40–49	3.70 ± 0.86	6.827	4	0.145	Not significant
	50–59	3.57 ± 0.93	6.827	4	0.145	Not significant
	≥60	3.53 ± 1.14	6.827	4	0.145	Not significant
Gender	Male	3.61 ± 1.00	1.417	2	0.492	Not significant
	Female	3.57 ± 0.91	1.417	2	0.492	Not significant
	Prefer not to say	3.39 ± 1.25	1.417	2	0.492	Not significant
Professional role	Psychiatrist	3.55 ± 0.99	3.289	5	0.655	Not significant
	Clinical psychologist	3.67 ± 0.97	3.289	5	0.655	Not significant
	Counselling psychologist	3.53 ± 0.98	3.289	5	0.655	Not significant
	Psychiatric nurse	3.57 ± 0.91	3.289	5	0.655	Not significant
	Psychiatric social worker	3.65 ± 0.89	3.289	5	0.655	Not significant
	Psychology student	3.51 ± 1.07	3.289	5	0.655	Not significant
Work setting	Private practice	3.66 ± 0.93	6.263	4	0.180	Not significant
	Hospital/mental health institution	3.63 ± 0.88	6.263	4	0.180	Not significant
	Academic/teaching institution	3.56 ± 1.03	6.263	4	0.180	Not significant
	Community mental health centre	3.47 ± 1.03	6.263	4	0.180	Not significant
	Others	3.08 ± 0.79	6.263	4	0.180	Not significant
Years of clinical experience	None	3.36 ± 1.12	25.950	4	<0.001	Very highly significant
	<5 years	3.66 ± 0.93	25.950	4	<0.001	Very highly significant
	5–10 years	3.64 ± 0.91	25.950	4	<0.001	Very highly significant
	11–20 years	3.57 ± 0.92	25.950	4	<0.001	Very highly significant
	>20 years	2.00 ± 1.37	25.950	4	<0.001	Very highly significant
Employment location	California	3.53 ± 0.99	88484.000	–	0.090	Not significant
	New Jersey	3.66 ± 0.92	88484.000	–	0.090	Not significant
Formal AI training	Yes	3.67 ± 0.92	78778.000	–	0.001	Highly significant
	No	3.43 ± 1.02	78778.000	–	0.001	Highly significant
Current AI use	Yes	3.65 ± 0.98	10.290	2	0.006	Highly significant
	No	3.46 ± 0.97	10.290	2	0.006	Highly significant
	Unsure	3.80 ± 0.73	10.290	2	0.006	Highly significant
Familiarity with AI	Not familiar	2.98 ± 1.12	64.394	3	<0.001	Very highly significant
	Somewhat familiar	3.51 ± 0.93	64.394	3	<0.001	Very highly significant
	Moderately familiar	3.68 ± 0.85	64.394	3	<0.001	Very highly significant
	Highly familiar	4.18 ± 0.91	64.394	3	<0.001	Very highly significant

Participants with more than 20 years of clinical experience demonstrated the lowest mean awareness score (2.00 ± 1.37), whereas higher mean scores were observed among participants with less than five years (3.66 ± 0.93), 5-10 years (3.64 ± 0.91), and 11-20 years (3.57 ± 0.92) of experience (lower mean scores were observed in the subgroup with more than 20 years of experience; however, this subgroup comprised only four participants and the findings should be interpreted cautiously). Awareness scores differed significantly between participants with and without formal training in AI, with participants reporting such training demonstrating higher mean scores (3.67 ± 0.92) compared to those without training (3.43 ± 1.02) (U = 78778.000; p = 0.001).

Additionally, awareness scores differed significantly based on current use or interaction with AI tools in professional practice (χ² = 10.290; p = 0.006). Participants who reported being unsure about AI use exhibited the highest mean awareness score (3.80 ± 0.73), followed by those who reported current use (3.65 ± 0.98), while those reporting no use demonstrated comparatively lower scores (3.46 ± 0.97). A highly significant difference in awareness scores was observed across levels of familiarity with AI concepts in mental healthcare (χ² = 64.394; p < 0.001). Mean scores increased progressively from the “not familiar” group (2.98 ± 1.12) to the “highly familiar” group (4.18 ± 0.91) (Table 7).

Bivariate differences in perception scores toward AI across participant characteristics

Perception scores toward AI in mental healthcare did not differ significantly by age, gender, or professional role (p > 0.05). However, significant differences were observed based on work setting, years of clinical experience, employment location, formal training in AI, and familiarity with AI concepts in mental healthcare (p < 0.05). Participants working in private practice demonstrated higher perception scores, while lower scores were observed in the subgroup with more than 20 years of clinical experience; however, this subgroup was very small (n = 4), and the findings should be interpreted cautiously. Participants from New Jersey and those who had received formal AI training reported significantly higher perception scores. Perception scores differed significantly across levels of familiarity with AI concepts, with mean values increasing progressively from the “not familiar” to the “highly familiar” groups. No statistically significant differences were observed based on current use or interaction with AI tools in professional practice (χ² = 5.954; p = 0.051) (Table 8).

**Table 8:** Bivariate differences in perception scores toward artificial intelligence across participant characteristics. All reported comparisons represent unadjusted, bivariate analyses. Values are expressed as mean ± standard deviation (SD). Comparisons across multiple groups were performed using the Kruskal–Wallis H test (#), while two-group comparisons were analyzed using the Mann–Whitney U test. χ² indicates the Kruskal–Wallis test statistic; df denotes degrees of freedom; U denotes the Mann–Whitney U statistic. Pearson’s chi-square test was not applied to ordinal Likert-scale outcome variables. A P-value < 0.05 was considered statistically significant. Additionally, findings related to participants with more than 20 years of clinical experience should be interpreted cautiously due to the very small subgroup size (n = 4).

Characteristic	Category	Mean ± SD	χ²/U value	df	p value	Interpretation
Age (years)	20–29	3.59 ± 0.93	7.226	4	0.124	Not significant
	30–39	3.67 ± 0.99	7.226	4	0.124	Not significant
	40–49	3.82 ± 0.91	7.226	4	0.124	Not significant
	50–59	3.61 ± 0.94	7.226	4	0.124	Not significant
	≥60	3.59 ± 1.04	7.226	4	0.124	Not significant
Gender	Male	3.65 ± 1.06	0.943	2	0.624	Not significant
	Female	3.73 ± 0.86	0.943	2	0.624	Not significant
	Prefer not to say	3.61 ± 0.69	0.943	2	0.624	Not significant
Professional role	Psychiatrist	3.69 ± 0.92	5.548	5	0.353	Not significant
	Clinical psychologist	3.69 ± 1.04	5.548	5	0.353	Not significant
	Counselling psychologist	3.76 ± 0.95	5.548	5	0.353	Not significant
	Psychiatric nurse	3.73 ± 0.83	5.548	5	0.353	Not significant
	Psychiatric social worker	3.61 ± 0.91	5.548	5	0.353	Not significant
	Psychology student	3.51 ± 1.02	5.548	5	0.353	Not significant
Work setting	Private practice	3.92 ± 0.80	18.085	4	0.001	Highly significant
	Hospital/mental health institution	3.75 ± 0.93	18.085	4	0.001	Highly significant
	Academic/teaching institution	3.59 ± 0.97	18.085	4	0.001	Highly significant
	Community mental health centre	3.63 ± 1.08	18.085	4	0.001	Highly significant
	Others	3.17 ± 0.39	18.085	4	0.001	Highly significant
Years of clinical experience	None	3.48 ± 1.07	13.932	4	0.008	Highly significant
	<5 years	3.76 ± 0.83	13.932	4	0.008	Highly significant
	5–10 years	3.74 ± 0.91	13.932	4	0.008	Highly significant
	11–20 years	3.66 ± 1.00	13.932	4	0.008	Highly significant
	>20 years	2.63 ± 1.46	13.932	4	0.008	Highly significant
Employment location	California	3.64 ± 0.93	86399.500	–	0.022	Significant
	New Jersey	3.76 ± 0.97	86399.500	–	0.022	Significant
Formal AI training	Yes	3.77 ± 0.88	81117.000	–	0.010	Significant
	No	3.56 ± 1.04	81117.000	–	0.010	Significant
Current AI use	Yes	3.74 ± 0.89	5.954	2	0.051	Not significant
	No	3.59 ± 1.04	5.954	2	0.051	Not significant
	Unsure	3.93 ± 0.66	5.954	2	0.051	Not significant
Familiarity with AI	Not familiar	3.19 ± 1.10	29.186	3	<0.001	Very highly significant
	Somewhat familiar	3.69 ± 0.95	29.186	3	<0.001	Very highly significant
	Moderately familiar	3.76 ± 0.85	29.186	3	<0.001	Very highly significant
	Highly familiar	4.00 ± 0.93	29.186	3	<0.001	Very highly significant

Ethical concerns related to AI

Comparison of mean ethical concern scores revealed no statistically significant differences across age groups (χ² = 8.914; df = 4; p = 0.063), gender (χ² = 3.868; df = 2; p = 0.145), or professional role (χ² = 5.303; df = 5; p = 0.380). Ethical concern scores were also comparable across employment locations (U = 90338.500; p = 0.244) and between participants with and without formal training in artificial intelligence within healthcare (U = 83331.500; p = 0.052), and this difference did not reach statistical significance. This difference did not reach statistical significance. In contrast, statistically significant differences in ethical concern scores were observed based on work setting (χ² = 10.269; df = 4; p = 0.036). Participants working in hospital or mental-health institutions (3.84 ± 0.92) and private practice (3.83 ± 0.89) demonstrated higher mean scores compared with those working in academic or teaching institutions (3.64 ± 1.04) and other settings (3.25 ± 0.75). Ethical concerns also differed significantly according to years of clinical experience (χ² = 15.342; df = 4; p = 0.004), with participants having more than 20 years of experience exhibiting the lowest mean score (2.56 ± 1.50). Interpretation of findings related to participants with more than 20 years of clinical experience is limited by the very small size of this subgroup (n = 4).

Ethical concern scores differed significantly across categories of current use or interaction with AI tools in professional practice (χ² = 9.652; df = 2; p = 0.008). Participants who were unsure about AI use reported the highest mean score (4.03 ± 0.74), followed by those reporting current use (3.79 ± 0.94), while those reporting no use demonstrated lower scores (3.62 ± 1.06). Ethical concern scores differed significantly across levels of familiarity with AI concepts in mental healthcare (χ² = 23.926; df = 3; p < 0.001), with mean scores increasing progressively from the “not familiar” group (3.48 ± 1.27) to the “highly familiar” group (4.13 ± 0.93) (Table 9).

**Table 9:** Bivariate differences in ethical concern scores regarding artificial intelligence across participant characteristics. All reported comparisons represent unadjusted, bivariate analyses. Values are expressed as mean ± standard deviation (SD). Comparisons across multiple groups were performed using the Kruskal–Wallis H test (#), while two-group comparisons were analyzed using the Mann–Whitney U test. χ² indicates the Kruskal–Wallis test statistic; df denotes degrees of freedom; U denotes the Mann–Whitney U statistic. Pearson’s chi-square test was not applied to ordinal Likert-scale outcome variables. A P-value < 0.05 was considered statistically significant. Additionally, findings related to participants with more than 20 years of clinical experience should be interpreted cautiously due to the very small subgroup size (n = 4).

Characteristic	Category	Mean ± SD	χ²/U value	df	p value	Interpretation
Age (years)	20–29	3.66 ± 1.05	8.914	4	0.063	Not significant
	30–39	3.70 ± 0.95	8.914	4	0.063	Not significant
	40–49	3.88 ± 0.95	8.914	4	0.063	Not significant
	50–59	3.63 ± 0.99	8.914	4	0.063	Not significant
	≥60	3.66 ± 1.23	8.914	4	0.063	Not significant
Gender	Male	3.74 ± 1.07	3.868	2	0.145	Not significant
	Female	3.71 ± 0.92	3.868	2	0.145	Not significant
	Prefer not to say	4.00 ± 0.72	3.868	2	0.145	Not significant
Professional role	Psychiatrist	3.81 ± 0.96	5.303	5	0.380	Not significant
	Clinical psychologist	3.82 ± 1.03	5.303	5	0.380	Not significant
	Counselling psychologist	3.67 ± 1.01	5.303	5	0.380	Not significant
	Psychiatric nurse	3.77 ± 0.85	5.303	5	0.380	Not significant
	Psychiatric social worker	3.77 ± 0.93	5.303	5	0.380	Not significant
	Psychology student	3.52 ± 1.12	5.303	5	0.380	Not significant
Work setting	Private practice	3.83 ± 0.89	10.269	4	0.036	Significant
	Hospital / mental health institution	3.84 ± 0.92	10.269	4	0.036	Significant
	Academic / teaching institution	3.64 ± 1.04	10.269	4	0.036	Significant
	Community mental health centre	3.63 ± 1.10	10.269	4	0.036	Significant
	Others	3.25 ± 0.75	10.269	4	0.036	Significant
Years of clinical experience	None	3.69 ± 1.14	15.342	4	0.004	Highly significant
	<5 years	3.89 ± 0.97	15.342	4	0.004	Highly significant
	5–10 years	3.75 ± 0.93	15.342	4	0.004	Highly significant
	11–20 years	3.66 ± 0.96	15.342	4	0.004	Highly significant
	>20 years	2.56 ± 1.50	15.342	4	0.004	Highly significant
Employment location	California	3.69 ± 1.01	90338.500	–	0.244	Not significant
	New Jersey	3.78 ± 0.96	90338.500	–	0.244	Not significant
Formal AI training	Yes	3.79 ± 0.93	83331.500	–	0.052	Not significant
	No	3.63 ± 1.08	83331.500	–	0.052	Not significant
Current AI use	Yes	3.79 ± 0.94	9.652	2	0.008	Highly significant
	No	3.62 ± 1.06	9.652	2	0.008	Highly significant
	Unsure	4.03 ± 0.74	9.652	2	0.008	Highly significant
Familiarity with AI	Not familiar	3.48 ± 1.27	23.926	3	<0.001	Very highly significant
	Somewhat familiar	3.62 ± 0.98	23.926	3	<0.001	Very highly significant
	Moderately familiar	3.83 ± 0.88	23.926	3	<0.001	Very highly significant
	Highly familiar	4.13 ± 0.93	23.926	3	<0.001	Very highly significant

## Discussion

The present study explored awareness, perceptions, ethical concerns, and perceived barriers and facilitators related to the adoption of AI among the mental-health workforce practicing in two US states. Overall awareness of AI among participants was moderate to high, with mean awareness scores ranging from 3.45 to 3.66 across awareness items and 63.2% of respondents reporting prior formal exposure or training in AI-related concepts. Despite this level of awareness, only 39.6% of participants reported current use of AI-assisted systems in clinical practice, indicating a clear gap between conceptual familiarity and real-world application. Similar patterns have been reported in previous studies. Topol described increasing clinician exposure to AI-driven diagnostic and administrative tools across healthcare systems while emphasizing that adoption often lags behind awareness due to implementation and trust-related barriers [3]. Jiang et al. also reported widespread awareness of AI applications among healthcare professionals, particularly in diagnostics and workflow optimization, but highlighted variability in clinical uptake across settings [4]. Additionally, Panch et al. noted that, although clinicians increasingly recognize the potential of AI within health systems, structural, ethical, and organizational constraints continue to limit routine integration into clinical practice [8].

This gap between awareness and adoption is consistent with observations by Longoni et al., who demonstrated that clinician resistance to medical AI often persists despite recognition of its potential benefits, largely due to concerns related to trust, accountability, and perceived loss of professional autonomy [6]. He et al. further emphasized that translation of AI from development to routine clinical use is frequently limited by challenges related to validation, scalability, and real-world implementation [14]. In addition, Kelly et al. underscored that without adequate institutional support, workforce training, and integration into existing clinical workflows, the clinical impact of AI technologies remains limited [13]. These findings align with emerging international evidence demonstrating a similar gap between AI awareness and practical application in healthcare settings. A cross-sectional study conducted in Jeddah, Saudi Arabia, reported substantial awareness of AI among healthcare workers but limited hands-on experience and formal training in AI applications [15]. Similarly, a multi-country survey from the United Arab Emirates demonstrated generally positive attitudes toward the role of AI in administrative and clinical processes, alongside inadequate training and educational opportunities for healthcare professionals [16].

Barriers to AI adoption reported in the present study further explain the observed gap between awareness and use. Lack of training, ethical or legal uncertainty, and limited resources were the most commonly cited barriers, while clear guidelines, professional education, and stronger clinical evidence were identified as key facilitators. Together, these findings highlight that the mental health workforce is broadly receptive to AI but requires structured support, ethical clarity, and practical integration strategies to translate awareness into responsible clinical adoption. Participants reported generally positive perceptions of AI in mental healthcare, particularly regarding its potential to enhance diagnostic accuracy, support early identification of relapse or risk behaviors, reduce administrative burden, and improve access to services. These findings align with existing literature indicating that clinicians tend to view AI as a complementary tool that can augment, rather than replace, clinical expertise [3,6,12].

An apparent paradox was observed in the present findings, wherein a substantial proportion of participants reported having received formal training in AI, yet a lack of training was simultaneously identified as the most common barrier to clinical adoption. This pattern may indicate that existing educational exposure is insufficiently aligned with real-world clinical implementation needs. Current training models may emphasize conceptual or technical aspects of AI without adequately addressing practical integration into clinical workflows, regulatory and legal considerations, or ethical governance frameworks. This interpretation is supported by the high proportion of participants identifying ethical and legal uncertainty as a major barrier, highlighting the need for training programs that extend beyond technical literacy to include applied, context-specific, and ethically grounded competencies.

Importantly, the perceived role of AI in improving access to mental health services received strong endorsement in the present study (mean = 3.75 ± 1.01). This finding is especially relevant in the US context, where persistent shortages of mental health professionals and geographic disparities continue to limit access to care. Thomas et al. documented substantial county-level shortages of mental-health providers across the US, highlighting the need for innovative approaches to service delivery [17]. AI-enabled screening tools, digital interventions, and remote monitoring systems may therefore be perceived by the mental health workforce as pragmatic adjuncts to address unmet needs rather than replacements for human care.

Ethical concerns and attitudes toward governance emerged as a prominent component of the present findings, with participants expressing strong concern regarding algorithmic bias, data privacy, informed consent, and the need for clear ethical guidelines prior to AI implementation. Among the ethical domains assessed, concerns related to bias in clinical decision-making demonstrated the highest mean score (3.81 ± 0.96), followed closely by concerns regarding privacy and data security (3.72 ± 0.99). These findings suggest that the mental health workforce remains particularly cautious about the downstream consequences of AI-assisted decisions, especially in psychiatric contexts where diagnostic ambiguity, comorbidity, and sociocultural influences are common. In addition, the high level of agreement on the need for informed consent before using AI tools (mean = 3.76 ± 0.99) highlights the mental health workforce's emphasis on patient autonomy and trust as essential components of the therapeutic relationship. It is important to note that this domain primarily captures perceived ethical concerns and endorsement of safeguards rather than objective ethical knowledge or competence.

Bivariate analyses indicated that awareness, perception, and ethical concern scores differed across levels of clinical experience, familiarity with AI concepts, and prior exposure to AI training. Lower scores were observed among participants in the highest experience category; however, the very small number of respondents in this group limits the stability and generalizability of this observation. Overall, these findings indicate that variation in AI-related attitudes appears more closely aligned with exposure to and familiarity with digital health technologies rather than clinical seniority alone, although causal inferences cannot be drawn. Participants reporting prior formal training in AI demonstrated higher awareness and perception scores in bivariate analyses, supporting the finding that 63.2% of respondents with prior training demonstrated more favorable attitudes toward AI use. However, training was not consistently associated with ethical-concern scores, indicating that technical instruction alone may not sufficiently address ethical preparedness. This underscores the importance of integrating ethics-focused content into AI training programs for mental health professionals and trainees.

Familiarity with AI concepts showed the strongest bivariate association with awareness, perception, and ethical concern scores, increasing progressively from participants who were not familiar with AI to those who were highly familiar. Notably, ethical-concern scores were the highest among participants reporting high familiarity, suggesting that greater exposure to AI is associated with higher ethical concern scores, although causality cannot be inferred. Work setting was also associated with differences in perception and ethical concern scores in bivariate analyses, with participants in private practice and hospital-based settings reporting higher mean scores compared with those in academic or other settings. These findings may reflect greater exposure to workflow-driven digital tools and operational demands in active clinical environments. In contrast, age and gender were not significantly associated with awareness, perceptions, or ethical concerns, reinforcing that professional and experiential factors play a more central role than demographic characteristics.

Several AI-based tools have already been deployed in mental healthcare, primarily for screening, symptom monitoring, psychoeducation, and early intervention. Conversational agents such as Woebot and Wysa use natural language processing and cognitive behavioral therapy-based frameworks to deliver real-time emotional support and psychoeducation and have demonstrated reductions in symptoms of depression and anxiety in controlled studies [18,19]. Tess, an AI-driven psychological support chatbot, has been used to provide mental health support across diverse populations and settings, showing improvements in psychological distress and user engagement [20].

In addition to chatbot-based interventions, emerging AI-enabled digital mental health platforms integrate machine learning with human-supported care models and predictive analytics to support mental well-being and early identification of mental health concerns. Such approaches commonly leverage digital phenotyping and behavioral data to inform screening, monitoring, and intervention strategies, as described in recent systematic reviews of AI applications in mental healthcare [21-23]. These developments further reinforce the importance of aligning technological innovation with clinician education, ethical safeguards, and appropriate clinical oversight.

Limitations and future directions

This study's cross-sectional design limits causal inference and captures perceptions of AI at a single time point. Data were collected through a self-administered online questionnaire, which may be subject to response and social desirability bias, and may overrepresent individuals with a greater interest in digital technologies. Additionally, the study was limited to two regions within the United States, which may restrict generalizability to other settings. Future research should employ longitudinal and mixed-methods designs to examine changes in clinician attitudes over time and to explore real-world ethical challenges associated with AI use in mental healthcare. Interventional studies assessing the impact of structured AI training and ethical education on clinical adoption and decision-making are also warranted.

Additionally, the extensive number of bivariate subgroup comparisons increases the possibility of false-positive findings, and some statistically significant results may not persist after more conservative correction for multiple testing. Moreover, the inclusion of both licensed professionals and trainees without stratified or adjusted analyses may have introduced heterogeneity in responses, which should be considered when interpreting subgroup comparisons.

An important limitation of the study is the non-availability of non-response rates, as the survey was disseminated through open electronic platforms and professional networks. Consequently, the total number of individuals who received the survey invitation could not be determined, which may limit the representativeness of the findings. Reporting of the online survey was guided by the CHERRIES (Checklist for Reporting Results of Internet E-Surveys) guidelines, although certain elements, such as response rate estimation, could not be fully addressed.

## Conclusions

The mental health workforce demonstrated generally positive awareness and perceptions of AI in mental healthcare, alongside prominent ethical concerns and limited clinical adoption. Differences in AI-related attitudes were observed across levels of familiarity with AI concepts, clinical experience, and prior exposure to AI training in exploratory, bivariate analyses, while demographic characteristics showed minimal variation. Interpretations related to participants in the highest experience category should be made cautiously due to small subgroup sizes. Overall, these findings underscore the need for context-specific education, strengthened ethical governance frameworks, and evidence-based implementation strategies to support the responsible integration of AI into mental healthcare practice.

## Appendices

Table 10 presents the questionnaire for assessing perceptions and ethical awareness of mental health professionals toward artificial intelligence.

**Table 10:** Questionnaire assessing perceptions and ethical awareness of mental health professionals toward artificial intelligence. Likert scale responses ranged from 1 (Strongly Disagree) to 5 (Strongly Agree). Additionally, barrier and facilitator response options were developed by the authors based on a review of prior literature and expert input; participants could additionally elaborate via open-ended items. The questionnaire was made by the authors.

Section	Item No.	Question/Statement	Response Format
Participant Information & Consent	1.1	I have read and understood the study information.	Yes/No
	1.2	I voluntarily consent to participate in this study.	Yes/No
	1.3	I am a qualified mental-health professional or currently in training.	Yes/No
Demographic Details	2.1	Age group.	20–29/30–39/40–49/50–59/≥60
	2.2	Gender.	Male/Female/Prefer not to say
	2.3	Professional role.	Psychiatrist/Clinical Psychologist/Counselling Psychologist/Psychiatric Nurse/Psychiatric Social Worker/Other
	2.4	Work setting.	Private practice/Hospital or mental health institution/Academic institution/Community mental health centre/Other
	2.5	Years of clinical experience.	<5 / 5–10 / 11–20 / >20 years
	2.6	Employment location.	California/New Jersey
	2.7	Formal training in AI in healthcare.	Yes/No
	2.8	Current use of AI tools in the professional setting.	Yes/No/Unsure
	2.9	Familiarity with AI concepts in mental healthcare.	Not familiar/Somewhat/Moderately/Highly familiar
General Awareness of AI	3.1	I am aware that AI is used in various areas of healthcare.	5-point Likert scale
	3.2	I have encountered AI-based tools relevant to mental health.	5-point Likert scale
	3.3	AI can assist in the early detection of mental-health conditions.	5-point Likert scale
	3.4	AI tools can reduce administrative workload for clinicians.	5-point Likert scale
Perceptions of AI in Mental Healthcare	4.1	AI can enhance diagnostic accuracy.	5-point Likert scale
	4.2	AI can support early prediction of relapse or risk behaviours.	5-point Likert scale
	4.3	AI-based virtual assistants can provide preliminary support to clients.	5-point Likert scale
	4.4	AI can improve accessibility of mental-health services.	5-point Likert scale
Ethical Awareness & Concerns	5.1	AI use raises concerns regarding privacy and data security.	5-point Likert scale
	5.2	Clear ethical guidelines are needed for AI in mental healthcare.	5-point Likert scale
	5.3	AI may introduce bias in diagnosis or decision-making.	5-point Likert scale
	5.4	Informed consent should be obtained before using AI tools.	5-point Likert scale
Barriers to AI Adoption	6.1	Barriers to adopting AI in mental-health practice.	Multiple response: lack of awareness, lack of training, ethical/legal uncertainty, high cost, resistance to change, other
Facilitators to AI Adoption	6.2	Factors that could facilitate AI adoption.	Multiple response: education and training, clear guidelines, clinical evidence, user-friendly tools, workflow integration
Clinical Use of AI	6.3	Have you used any AI-assisted system in your clinical work?	Yes/No
	6.4	If yes, please specify the AI tool/system used.	Open-ended (optional)
Open-Ended Reflection	7.1	Most important consideration for the responsible use of AI in mental-health practice.	Open-ended
Acknowledgment	—	Thank you for your time and valuable participation.	—
